# User and researcher collaborations in mental health in low and middle income countries: a case study of the EMPOWER project

**DOI:** 10.1186/1756-0500-7-37

**Published:** 2014-01-14

**Authors:** Esha Gupta, Bayard Roberts

**Affiliations:** 1 London School of Hygiene and Tropical Medicine, Keppel St, Bloomsbury WC1E 7HT, UK; 2 Health Systems and Policy, European Centre on Health of Societies in Transition London School of Hygiene and Tropical Medicine, 15-17 Tavistock Place, London WC1H 9SH, UK

**Keywords:** Mental health, Research, User

## Abstract

**Background:**

Increasing recognition has been given to the interaction of users and researchers in shaping the perspective and practice of mental health care. However, there remains very little evidence exploring how this interaction works, particularly in low and middle income countries. The aim of this study was to explore experiences of how users and researchers worked together to communicate research, using a case study of the EMPOWER project.

**Methods:**

The study followed a case-study approach. EMPOWER was a project that sought to strengthen the capacity of user organizations in India, Kenya, Nepal and Zambia by encouraging user-researcher collaborations to communicate research findings in the four countries. A qualitative research method was applied for this study, with semi-structured interviews conducted with seven people: two researchers, one communications developer, and four user group members (one from each of the four countries). Data were analyzed using thematic analysis.

**Results:**

The findings indicated positive perceptions of the collaboration between researchers and users. Key themes were partnership and support, the value of the personal experience of users and their knowledge of the target audiences, and empowerment. Key challenges related to differences in levels of education and technical knowledge and the lack of payments to users.

**Conclusions:**

This exploratory study provides insight to help understand collaborative processes for communicating mental health research. It highlights many positive outcomes from the EMPOWER collaboration but also highlights the need for more in-depth research on this issue.

## Background

There has been an increasing move away from the traditional view of mental health care users as “passive recipients” [1], with more attention given to users in terms of what services they need and want, including in the field of mental health research [2]. This has included increasing interaction of mental health care users and researchers in shaping the perspectives and practice of mental health care, in the belief that users can provide fresh insights ‘from the inside’ [3], that users as researchers bring a ‘real world’ validity to research [4], leading to more effective research and subsequently more accessible, appropriate and acceptable services, better public understanding of mental health, and reduced stigma against people with mental disorders [5-11].

A key element in user-researcher collaboration is also involving users in the communication of research findings to users, their carers and families, and to communities more broadly. However, there is limited information on mental health user and research interaction and this existing research appears mainly limited to the United Kingdom and United States [3,5-7,9,12]. This is particularly the case with user-researcher collaboration in the dissemination of research findings to the general public.

The aim of this study was to explore experiences of how users and researchers worked together to communicate research, using a case study of the EMPOWER project. The objectives were to: (i) explore the roles and interaction of users and researchers; and (ii) explore the perceived benefits and challenges of the collaboration.

The EMPOWER project was established in 2010, with funding from the Wellcome Trust. It sought to develop public engagement materials to communicate mental health research findings to local audiences in India, Kenya, Nepal and Zambia. The project partners were Sangath in India, the London School of Hygiene and Tropical Medicine in the United Kingdom, the Richmond Fellowship Society in India, the Users and Survivors of Psychiatry Group in Kenya, the Nepal Mental Health Foundation, and the Mental Health Users Network of Zambia. Users and researchers jointly developed locally appropriate communication products through a process a regular face-to-face meetings and telephone and video Skype calls. The communication products included posters, songs, theatre shows, websites, videos and television documentaries. These sought to respond to key research findings by addressing issues such as: early diagnosis and referral; raising community awareness about common mental disorders; de-stigmatising mental disorders; and improving understanding, support and care for people with mental disorders. Further details on the project and examples of the communications materials can be found at [13].

## Methods

The study followed a case-study approach and a qualitative research design. Semi-structured interviews were conducted via Skype by EG with seven respondents. These were with the four user group leaders (referred to hereon as ‘users’) in the four EMPOWER countries of Kenya, Zambia, Nepal, and India in order to gain the perspective of users. User group leaders were mental health users selected by the EMPOWER country partners and they were responsible for communicating directly with EMPOWER researchers to develop the communication products for their countries. One semi-structured interview was also conducted with a communications consultant in South Africa who contributed to the development of the EMPWOWER communication products. Two semi-structured interviews were also conducted with the lead researchers who were mental health academics based in India and Australia.

Three separate topic guides were created for the researchers, users, and the communications consultant in South Africa. The common core contents of the topic guides included: respondents background and personal experiences of being involved in mental health related activities; their roles in the Empower study; past experiences of collaborations between users and researchers; perceptions on the process and experience of being involved in the EMPOWER study; perceptions on communications and relations between members, including issues of equality and hierarchy; perceived benefits and challenges of the EMPOWER study process; overall impressions and recommendations.

All interviews were conducted in English and their duration ranged from 30 to 90 minutes. After each call, the recordings were played back and the interviews transcribed verbatim. The data were analyzed using thematic coding techniques, with themes coded and analyzed within the framework of the two study objectives. The key themes that emerged for objective one were partnership and support. The key themes that emerged for objective two were the value of the personal experience of users, their knowledge of the target audiences for the communications materials, and empowerment, with challenges including differences in educational backgrounds and technical knowledge and the role of payments to users.

All respondents were provided with an information sheet and an informed consent document. Respondents gave written informed consent via email and also verbal consent before the interview. All interviews were treated as confidential and anonymous, with no names attached to any transcripts. Ethical approval was provided by Ethics Committee of the London School of Hygiene and Tropical Medicine.

## Results

The first main objective explored the roles and interaction of users and researchers. Two key interlinked themes related to this objective were those of partnership and support between the users and researchers. Researchers in EMPOWER were largely responsible for developing the overall project framework, while the users provided input and suggestions and were a part of the partnership from the start. Users recognized that some of the work was more appropriate for the researchers because of their research expertise. At the same time, researchers appreciated that while they could contribute to EMPOWER with their research skills, users were able to bring personal experience and knowledge that could be translated into practical communication products. One user said:

“We benefited because the researchers used to bring the experience of years of research, and we used to feel like our experience could work in real life and through that collaboration even we benefited.”

Users had the general view that while their experiences in EMPOWER were a collaborative effort, they were allowed to independently decide what types of communication products they wanted to develop for their countries and how they were going to develop them. One user stated:

“We discussed what we wanted to do and it was innovative and experimental, no one gave us ideas to impose…we were free to suggest what we wanted to do. There was no fixed role or pre-imposed role…our roles were decided by ourselves.”

One researcher described the collaboration as a consultative approach, with users leading the product development and consulting with researchers for their suggestions or guidance, noting it was based on “a lot of mutual support and suggestions.” One of the users noted:

“I would take guidance from him [a researcher] whenever I had to. We had Skype meetings for all the EMPOWER groups…He was guiding because he left the implementation part to us but he was keeping track of how we were doing things and what help we needed. He was monitoring. Any time I got stuck, I could call on him.”

One user contrasted the positive process of EMPOWER with their previous experiences:

“Often you will find mental health users on one side and researchers on the other side and no communication between the two due to high levels of stigma…users are singled out…to change this, it needs to be collaboration.”

The second main study objective explored the perceived benefits and challenges of the collaboration. A key theme relating to the benefits of the collaboration was the value of the personal experience of users and their knowledge of the target audiences for the communications materials. The users understood the issue of mental health through personal experiences. One user explained that user knowledge was like an “untapped resource that was not available in books”. The user stated that EMPOWER provided a platform that actually utilized these resources and allowed users to share their first hand knowledge and experiences on mental health. The personal challenges, abuse, ostracism, and stigmatism faced by users in their lives helped them understand what types of EMPOWER products needed to be created to advocate for mental health in their communities. One of the respondents said that because of her personal experiences of involuntary lock ups and abuse because of her mental disorders, she understood that people suffering from mental disorders are often portrayed very aggressively, which in turn affects the way they are viewed in their community. The respondent therefore made the argument to “break away from this aggressive portrayal because people already related mental illnesses with aggressive, violence, and crime” and encouraged to change the character and focus on educating the community on true causes, symptoms and effects of mental disorders.

One of the users mentioned that traditionally only academic researchers were perceived as being experts in mental health but with EMPOWER users are considered experts because it is recognized that they have real experience. A researcher noted:

“The experience of working together collaboratively and the openness and respect user groups have towards biomedical research is very important because the relationships between user groups and research and medicine is a mixed one. In different contexts, there is different relationships and is often based on the history of biomedicine.”

In working with the users and learning from them, one researcher noted how it would also influence their future research:

“It is centrally important to work with user communities not in just communicating research but also working with them to understand what the research questions may be. EMPOWER did not start off with that goal, but in spending a year and a half in working with colleagues of mine, I began understanding much better the everyday challenges and lives of people affected by mental illness in different contexts and I am pretty sure, at least I would like to think, I have been profoundly influenced by that, in my own thinking about the sorts of questions I want to address as a researcher in the future.”

The importance of knowing the target audience for the communications materials was a recurring theme. Because the users in EMPOWER were from the communities that they had created the communication products for, they knew their target audiences well and what products could be most effective for mental health advocacy. Almost all of the users mentioned how they felt they had a strong knowledge of the types of product to create and their content to ensure their effectiveness with the target audiences. This also resulted in a more constructive portrayal of people with mental health care problems. One user noted how:

“There are messages that are not rightly disseminated in films and media in showing positive mental health; mental health is always shown in a negative way. Here the messages were sought from people who were actually affected.”

Another key theme related to the benefits of the project was the sense of empowerment among users. Several users noted how the additional confidence and acquisition of new skills supported them in their own work. Due to the stigma from mental disorders, people with disorders are often marginlised and viewed as having little contribution to make to society. EMPOWER helped to demonstrate their value and productivity, which could help reduce stigma towards people with mental disorders. One user explained that when people in communities would see the products and understand that they are made by people who have mental disorders, users would no longer be viewed “useless”. In addition, users themselves felt empowered by having produced the materials. One user noted:

“I was very much encouraged because there is someone here who thinks we are capable of contributing to research, that is a major empowerment feeling.”

A number of challenges in the collaboration were also raised. While users and researchers for the most part felt comfortable with each other, there was some discomfort in the collaboration relating to differences in educational level and technical knowledge. One respondent noted how:

“They [researchers] have high education and they have obtained a high level of knowledge in global mental health. I was a bit uncomfortable, even though I would love to have that knowledge, I have not been exposed to it.”

One user explained there was also a discomfort in the collaboration due to unfamiliarity of working with researchers. The user noted:

“In my country, it is very rare that researchers would interact with a person with a mental illness…I have mental illness. There has never been a mix with mental health professionals and users. That needs to change.”

An additional challenge was the different approaches to the work followed by users and researchers. This led to discussion on what is feasible and reasonable to expect from people with no formal research training. One researcher commented:

“I don’t know if it was a big challenge but as a researcher there is a need to be systematic and do things a certain way if you want to get published for example, but that’s not a [user’s] priority necessarily. For a user, he just wants to get the product developed and ready and appropriate.”

The same researcher also commented that care needs to be taken on how much technical research-oriented detail the users should be exposed because of their limited research training.

The voluntary basis for the work of the users was also raised, with one user suggesting that if there was a bigger budget then users could have transitioned from volunteers to paid workers which would have legitimized and strengthened their roles in EMPOWER. The user noted if EMPOWER had plans to expand:

“The next phase should have a big budget and our work should not be as volunteer but also as professional to engage in EMPOWER.”

## Discussion

This study explored how EMPOWER researchers and users worked together to communicate mental health research findings. Key themes were partnership and support, the value of the personal experience of users and their knowledge of the target audiences, empowerment, and the challenge related to differences in levels of education and technical knowledge and the role of payments to users.

There is increasing recognition of the value of working with users in the field of mental health as ‘experts by experience’ because they have first hand experiences of mental health, stigma and discrimination, experiences of treatments (and lack of access to treatments), and the communities in which they live [1,3-7,12,14,15]. It has been proposed that this interaction between users and researchers typically involves three levels of interaction: consultative, user-led and collaborative [3,6,9]. Consultation is where users have limited sharing power and are consulted about a piece of research that requires users to share their opinions; user-led research and user-controlled research is where users control all stages of the research process and researchers provide additional support; and collaboration reflects an active and ongoing partnership between users and researchers where both groups share decision-making powers and are equally involved in the research process. The findings from this study suggest a mixed approach of collaborative and user-led styles was followed in the EMPOWER project, with users leading the direction and development of the materials, and researchers leading on the initial project design and then providing support and advice thereafter. This is in contrast to observations of other collaborations where user involvement appeared to remain more limited to sharing information rather than a more fundamental partnership or collaboration [14,16,17].

The study findings highlighted the sense of empowerment among users from the collaboration, particularly in areas of self-esteem, confidence, and motivation to expand their professional lives and a greater sense of social inclusion. These benefits are also reflected in other studies on mental health user-researcher collaborations which also highlight benefits such as feeling more included in societies where they may have previously felt excluded because of stigma, discrimination, unemployment, and limited social roles and networks [5-7].

The findings highlight challenges related to differences in educational backgrounds and technical expertise. This is reflected on a larger scale in other studies which have noted that while researchers are supportive of user involvement, there can be discrepancies between actual practice of user involvement and the expressed support [18]. Evidence has also suggested that some researchers believe they have a more ‘scientific’ way and users have a more ‘social’ way of thinking and working which can cause differences in approach and working relationships [19]. Studies have also noted differences about what is considered feasible and reasonable to expect from users when they do not have formal research training. While the different perspective provided by users and researchers can be a creative and rewarding process, it can create divergent expectations. In some situations, it has also been noted that some professionals find it difficult to regard users as experts in the field and are thus resistant towards user involvement [7].

Interestingly, none of the users in this study mentioned issues of power, despite its potentially significant role in user-researcher relations [3,20,21]. It is recognized that respondents, particularly users, could have, consciously or subconsciously, felt inhibited to talk about any imbalance of power in order to maintain positive relationships with EMPOWER colleagues and associations of the EMPOWER project more broadly. Differences in education, wealth, and socio-cultural backgrounds could potentially also have limited respondents’ perceptions of power imbalances and their willingness to express such imbalances. It would be beneficial for future research to explore the power dynamics of such collaborations in more depth.

The voluntary nature of the user involvement in EMPOWER was raised by some respondents. If such collaborative programmes are to be scaled up, formal payments may need to be considered. Studies from elsewhere argue that that collaborative activities should include payment as it helps recognizes the time and expertise provided by users and validate and professionalises their contribution [6,7,22]. Trivedi and Wykes note that it is important to have a contract to formalize the partnership between researchers and users [12]. This should include how both groups will be involved in the research, what is expected from users and researchers, and should also reflect how users will be protected.

### Limitations

This was a small exploratory study with a number of limitations. The study consisted of only seven interviews and so provides only a limited number of perspectives. This was because only the four user group leaders, the two researchers and the communications consultant were directly involved in the collaboration. The small number of respondents reflects the small scale of EMPOWER which also meant that respondents knew each other and so may have felt uncomfortable sharing their true experiences and opinions for fear of damaging personal relationship and any future possible collaborations. There could also have been a power imbalance between the respondents and the researcher who conducted the interviews (EG) for this paper, particularly as EG was from the same institution as the Principal Investigator of EMPOWER. However, the confidential and anonymous nature of the interviews was reaffirmed by the researcher, along with the importance and value of objectively and critically appraising the collaborative process. The fact that some critical comments (albeit mild) were made by users suggests a degree of willingness to speak openly but this is difficult to determine objectively. The use of remote interviews through Skype and telephone may have meant that points and messages could have been missed which may not have been the case with face-to-face interviews where a greater rapport and understanding could have been established. Lastly, the focus of EMPOWER on the dissemination of research materials also means that this study cannot shed light on broader collaborations between researchers and users in the full research cycle of issue identification, research design setting, data collection and analysis, and disseminating and communicating research findings. The inherent limitations with our study reflect some of the difficulties of evidencing the influence of service user involvement with mental health research [23]. Alternative methods of analysis such as Interpretive Phenomenological Analysis could be useful methods to help improve understanding of user and researcher perspectives on the experiences, meanings and values related to their collaborations [24].

## Conclusions

There appears to be a growing awareness of the value of users and researchers collaborating together, but this remains at a rather nascent stage in the field of mental health, particularly in low and middle income countries. This exploratory study provides insight to help understand collaborative processes for communicating mental health research. It highlights many positive outcomes from the EMPOWER collaboration but also highlights the need for more in-depth research to systematically evaluate user-researcher collaboration in low and middle income countries and help maximize its potential value.

## Competing interests

This study’s authors are based at the London School of Hygiene and Tropical Medicine which was one of the partners in the EMPOWER project. The Principal Investigator of EMPOWER was also based at the London School of Hygiene and Tropical Medicine. However, the authors had no knowledge or involvement in the EMPOWER project prior to this research study. The Principal Investigator of EMPOWER also had no involvement in the content of this manuscript.

## Authors’ contributions

EG led the design of the study, the data and analysis, and writing of the manuscript. BR contributed to the study design and writing of the manuscript. Both authors read and approved the final manuscript.
